# Differences in serum concentrations of per-and polyfluoroalkyl substances by occupation among firefighters, other first responders, healthcare workers, and other essential workers in Arizona, 2020–2023

**DOI:** 10.1038/s41370-025-00753-7

**Published:** 2025-03-06

**Authors:** Cedar L. Mitchell, James Hollister, Julia M. Fisher, Shawn C. Beitel, Ferris Ramadan, Shawn O’Leary, Zhihua Tina Fan, Karen Lutrick, Jefferey L. Burgess, Katherine D. Ellingson

**Affiliations:** 1 Epidemic Intelligence Service, CDC, Atlanta, GA, USA.; 2 Pima County Health Department, Tucson, AZ, USA.; 3 Mel and Enid Zuckerman College of Public Health, University of Arizona, Tucson, AZ, USA.; 4 Statistics Consulting Laboratory, BIO5 Institute, University of Arizona, Tucson, AZ, USA.; 5 Environmental and Chemical Laboratory Services (ECLS), Public Health & Environmental Laboratories (PHEL), New Jersey Department of Health (NJDOH), Ewing, NJ, USA.; 6 College of Medicine - Tucson, University of Arizona, Tucson, AZ, USA.

**Keywords:** Per-and polyfluoroalkyl substances, Occupational health, Firefighting, Healthcare workers

## Abstract

**BACKGROUND:**

Certain occupations have greater risk for per- and polyfluoroalkyl substances (PFAS) exposure because of PFAS use in occupation-associated materials.

**OBJECTIVE:**

We sought to assess whether PFAS concentrations differed by occupation among certain Arizona workers and whether concentrations differed over time by occupation.

**METHODS:**

Serum concentrations for 14 PFAS were measured among 1960 Arizona Healthcare, Emergency Responder, and Other Essential Worker Study participants. Samples were collected at enrollment and periodically during July 2020–April 2023. Occupational categories included firefighters, other first responders, healthcare workers, and other essential workers. We fit multilevel regression models for each PFAS to estimate differences in geometric mean concentrations or odds of PFAS detection at enrollment by occupational category. For participants with ≥1 serum sample, we evaluated for yearly longitudinal differences in PFAS concentrations by occupational category. We used other essential workers for comparison, and adjusted for age, sex, race and ethnicity, year, and residential county.

**RESULTS:**

Adjusting for covariates, firefighters had higher perfluorohexanesulfonic acid (PFHxS), branched and linear perfluorooctanesulfonic acid (PFOS), and perfluoroheptanesulfonic acid (PFHpS) concentrations than other essential workers (geometric mean ratios 95% CIs: 1.26 [1.11–1.43]; 1.18 [1.06–1.32]; 1.19 [1.08–1.31]; and 1.19 [1.01–1.39], respectively). Healthcare workers had higher odds of detection of branched perfluorooctanoic acid (Sb-PFOA) and perfluorododecanoic acid (PFDoA) than other essential workers, adjusting for covariates (odds ratios 95% CIs: 1.35 [1.01–1.80]; 2.50 [1.17–5.34], respectively). During the 3-year study, we detected declines in PFAS concentrations among other essential workers; few longitudinal differences in concentrations by occupation were detected.

**IMPACT STATEMENT:**

Using data from a large prospective cohort of frontline workers in Arizona, we compared serum concentrations of 14 per-and polyfluoroalkyl substances (PFAS) among firefighters, other first responders, healthcare workers, and other frontline essential workers. We found that firefighters have higher concentrations of certain PFAS chemicals and the odds of detecting other PFAS chemicals are higher among healthcare workers compared with people in other occupations. Our findings highlight the importance of further action to reduce PFAS exposure within highly exposed occupational groups, such as firefighters, and the need to expand evaluation of exposure among other occupations, including healthcare workers.

## INTRODUCTION

Per-and polyfluoroalkyl substances (PFAS) are a class of thousands of synthetic chemicals used in a wide range of products and materials because of their stain-, water-, and flame-resistant properties. Because of their highly fluorinated aliphatic chemistry, PFAS exhibit limited degradation over time and have been detected widely as environmental contaminants in the United States from manufacturing processes, military installations, and disposal of PFAS-containing products [1, 2]. Exposure to PFAS among the U.S. population is typically through ingestion of contaminated water or food, and through contact with PFAS-containing products. PFAS can bioaccumulate in tissues after initial absorption [3] and remain detectable in human sera long after exposure, with the elimination half-life varying by specific PFAS chemical [4–6]. Elevated serum concentrations of certain PFAS have been associated with increased risk for kidney and testicular cancers, increases in cholesterol levels, lower antibody response to certain immunizations, pregnancy-induced hypertension and preeclampsia, small decreases in infant birth weight, and changes in liver enzymes [7, 8]. Because of associated adverse health effects, production in the United States of certain PFAS has ceased [1, 2, 9] and others are being phased out. Given that PFAS were being imported into the United States, the Environmental Protection Agency has promulgated PFAS importation reporting and recordkeeping requirements under the Toxic Substances Control Act [10]. Reductions in PFAS use and importation correlate with declining serum concentrations of PFAS in persons over time [1]. However, an estimated 99% of the U.S. population still have detectable levels of certain PFAS, and persons in occupations with more frequent exposure to higher concentrations of PFAS-containing materials have been shown to exhibit higher serum concentrations of some PFAS [5, 11, 12].

Workers in certain occupations have historically been exposed to high concentrations of PFAS through routine work duties. These occupations have included workers in PFAS production facilities, ski waxers, and firefighters [12, 13]. Occupational exposure to PFAS among firefighters has been a focus of study in recent years. As an occupational group, firefighters might have frequent contact with high concentrations of PFAS-containing materials during firefighting activities and have had elevated concentrations of PFAS detected in their sera relative to the general population [13, 14]. Historical exposure to fluorosurfactant-based aqueous film forming foam (AFFF) has led to elevated concentrations of perfluorohexanesulfonic acid (PFHxS), perfluorooctanesulfonic acid (PFOS), and perfluorooctanoic acid (PFOA) among firefighters who were employed during its use [5, 13, 15, 16]. Firefighting turnout gear remains a source of exposure to PFAS-containing moisture barriers embedded in jackets, pants, gloves, and boots [5, 13, 14, 17, 18]. Certain PFAS chemicals and integration of PFAS into turnout gear have changed over the past decade to reduce exposure [5, 14, 18]; howeve,r PFAS remain detectable in turnout textiles [18] and the magnitude of exposure from turnout gear requires further study. In certain locations, drinking water sources [6, 13] and food that is grown [19] around fire stations have been contaminated by historical use of AFFF. Inhalation of smoke and particulate matter from PFAS-containing household or structural materials during fires might be another source of exposure [5, 13, 20]. In the United States and internationally, numerous studies have reported elevated concentrations of PFHxS [15, 21–24], PFOS [21–23], PFOA [21, 23–25], and perfluorononanoic acid (PFNA) [15, 23, 25] among municipal firefighters when compared with population representative samples [23], or other nonexposed workers [15].

Increased awareness of PFAS exposure and elevated PFAS serum concentrations among firefighters has led to interventions to reduce sources of exposure and contact with PFAS-containing materials. Interventions have included substitution of PFAS in AFFF with nonfluorinated compounds [9, 17] and exploration of short-chain PFAS or alternative nonfluorinated compounds for turnout gear [5, 14, 17], protocols for handling and washing turnout gear to minimize dermal exposure [5, 14, 17], and use of self-contained breathing apparatuses to reduce inhalation of smoke and debris from PFAS-containing materials during fires [5, 17]. As dynamics of PFAS exposure pathways among firefighters continue to change, gaps remain in understanding how concentrations of legacy PFAS and other PFAS might change among firefighters.

PFAS exposure among other occupational groups of frontline workers remains largely unknown. Studies of PFAS exposures among workers have focused on occupational groups with known exposure to PFAS, such as workers in PFAS production facilities, ski waxers, and firefighters [5, 12, 13]. Given the widespread use of PFAS in textiles, medical devices and equipment, packaging, radiograph film, and other materials [11, 26], workers in certain occupations might be routinely exposed to discrete sources of PFAS, yet these potential occupational exposures remain uncharacterized. To address these gaps, we sought to assess whether PFAS concentrations differed among firefighters, other first responders, healthcare workers, and other essential workers in Arizona, and if differences changed over time. We used data from the large, prospective Arizona Healthcare, Emergency Response, and Other Essential Workers (AZ HEROES) cohort study.

## METHODS

### Study Population

The AZ HEROES cohort study was implemented to study SARS-CoV-2 infection and immunity in Arizona’s frontline workers and has been described in detail elsewhere [27]. In brief, AZ HEROES enrollment began July 27, 2020, and data collection for the study concluded April 15, 2023. Blood specimens were requested to be submitted within five days of enrollment. After enrollment, participants were asked to submit blood samples at approximately 3-month intervals, and from 14 to 28 days after each dose of SARS-CoV-2 vaccine, and approximately 28 days after a polymerase chain reaction-confirmed SARS-CoV-2 infection. Frontline occupations were categorized as firefighters, other first responders (e.g., emergency medical services, law enforcement, correctional officers), healthcare workers (e.g., clinical providers with direct interaction with patients in inpatient, outpatient, or residential settings), and other essential workers (e.g., retail, education, utilities, and/or government workers) [27].

### Ethical approval

This activity was determined to meet the Centers for Disease Control and Prevention (CDC) definition of research [45 CFR 46.102(l)] involving human subjects [45 CFR 46.102 (e)(1)] and Institutional Review Board (IRB) approval was provided by University of Arizona IRB (protocol #2006729444). All study participants provided written, informed consent for the parent study and secondary analyses for PFAS.*

### Measurement of Serum PFAS Concentrations

Serum PFAS concentrations were measured for a subset of AZ HEROES participants who had at least one whole blood specimen collected during July 27, 2020–April 15, 2023, and consented to receive their PFAS results. PFAS testing was conducted by the New Jersey Department of Health (NJDOH) [28] in accordance with CDC method #6304.09 [29]. Initially, eighteen PFAS were evaluated for in blood specimens. PFAS were included in the analysis if at least 10% of participants had a detectable value. The following PFAS were included: perfluorohexanesulfonic acid (PFHxS), branched and linear perfluorooctanesulfonic acid (Sm-PFOS; n-PFOS), branched and linear perfluorooctanoic acid (Sb-PFOA; n-PFOA), perfluoroheptanesulfonic acid (PFHpS), perfluorodecanoic acid (PFDA), perfluoroundecanoic acid (PFUnA), PFNA, perfluorododecanoic acid (PFDoA), 2-(N-methyl-perfluorooctane sulfonamido) acetate (Me-PFOSAA), perfluorooctanesulfonamide (PFOSA), perfluorobutanesulfonic acid (PFBS), and perfluoroheptanoic acid (PFHpA). Except for Sm-PFOS, n-PFOS, Sb-PFOA, and n-PFOA, all PFAS had isomers measured together. PFAS were selected for measurement based on availability of assays. The limit of detection (LOD) for certain PFAS assays changed during the study period (Supplemental Table 1). For measures below the LOD, a value equal to the LOD divided by the square root of two was imputed in accordance with previously published methods [30]. Availability of assays for Sm-PFOS, n-PFOS, Sb-PFOA, n-PFOA, PFHpS, and PFDoA changed over time. For participants who were missing a PFAS result for one of these six PFAS for their first serum specimen and had a later specimen, PFAS results and the collection date for the later specimen were used as their first specimen for this dataset. All PFAS included in the analysis are listed in Supplementary Table 1, including the proportion of samples above the LOD overall and stratified by occupation.

A threshold of 50% of measured results above the LOD was used to determine subsequent analyses. For PFAS with more than 50% of results above the LOD, PFAS outcomes were coded as continuous, and geometric means and geometric mean ratios were estimated. For all other PFAS which did not meet this threshold, outcomes were coded as binary detectable or not detectable, and odds of detection were estimated.

Crude geometric mean serum PFAS concentrations for all PFAS with more than 50% of results above the LOD were estimated by occupational group and compared with PFAS serum concentrations from adults in the most recent National Health and Nutrition Examination Survey (supplementary material).

### Cross-sectional regression analysis of PFAS concentrations by occupation

To evaluate cross-sectional associations among occupations and each PFAS at enrollment, we fit a series of multilevel regression models. For PFAS with more than 50% of participant samples above the LOD, we fit a linear multilevel regression model to estimate differences in geometric mean PFAS concentrations. For the remaining PFAS with 50% or fewer participant samples above the LOD, we estimated the odds of PFAS detection using a logistic multilevel regression model. For both models, a random intercept was specified per county of residence to account for correlation in PFAS exposure by location. Fixed effect covariates included occupation (i.e., firefighter, other first responder, healthcare worker, and other essential worker), age, self-reported sex (male or female), self-reported race and ethnicity (non-Hispanic White, non-Hispanic Black, non-Hispanic Asian, and Hispanic), and year of enrollment (as a categorical variable for number of years from 2020). All occupation comparisons were conducted using other essential workers as the referent group because they were most representative of the working public and were derived from the same sampling frame as other occupations in the parent study. All other covariates were identified as potential confounders using a directed acyclic graph. To evaluate sex as a potential effect modifier, a sex-stratified analysis was conducted and summarized in the supplement. For the linear multilevel regression models, the Kenward-Roger approximation for fixed effect standard errors and error degrees of freedom was used to account for differing degrees of freedom across levels of the random effect for county of residence [31].

### Longitudinal change in PFAS concentrations by occupation

To evaluate differences in PFAS concentrations over time by occupation, we fit a series of longitudinal linear multilevel regression models. Models were fit to PFAS with more than 50% of participant samples above the LOD to allow for a linear regression analysis. A random intercept was specified per participant to account for correlation in PFAS concentrations by participant. A variable for time was included as a continuous measure in years between the date of each participant’s first specimen collection and the date of the consecutive specimen collection. The origin was set to equal zero on the date of the first specimen collection for each participant. Time was included as an interaction term with occupation to flexibly model differences in time-varying PFAS measures among occupational groups. Other fixed effect covariates were coded the same as for the cross-sectional models and included occupation with other essential workers as the referent group, age, self-reported sex, self-reported race and ethnicity, and year of enrollment. The county of residence was modeled as a fixed effect to accommodate small cell counts. Kenward-Roger approximation was again used to account for differing degrees of freedom across levels of the random effect for a person.

All analyses were conducted, and figures produced in R (version 4.3.0; R Development Core Team) using lme4 [32] and ggplot2 [33] packages.

## RESULTS

### Characteristics of the study population

During the study, ≥1 PFAS measures were available for 1960 participants, including 280 (14%) firefighters, 159 (8%) other first responders, 787 (40%) healthcare workers, and 734 (37%) other essential workers (Table 1). Demographic and enrollment characteristics were similar among occupation groups except for the sex distribution, which showed fewer females among firefighters (18% female) and other first responders (43% female), compared with other essential workers (67% female), and healthcare workers (78% female). More than one serum sample analyzed for PFAS was available for 35% of all participants with a range of two to eight available samples. Collection times for later samples ranged from 2 weeks to 2.6 years after study enrollment.

The number of participants with available PFAS results changed for some PFAS measures because of differences in the availability of laboratory assays over time. Of 1960 participants with any PFAS measure at enrollment, 149 were missing measures for Sm-PFOS, n-PFOS, Sb-PFOA, n-PFOA, and PFHpS, and 99 had results from a later specimen collection date that was used in this analysis as their first specimen collection date. In total, 1811 participants had available results for Sm-PFOS, n-PFOA, Sb-PFOA, n-PFOA, and PFHpS. For PFDoA, 1712 participants were missing a measurement at enrollment resulting in 248 participants with a result for PFDoA (Supplementary Table 1).

### PFAS detectability

Eight of 14 PFAS measures had quantified results above the limit of detection for more than 50% of participants and were analyzed as continuous measures. These PFAS included PFHxS, Sm-PFOS, n-PFOS, n-PFOA, PFHpS, PFDA, PFUnA, and PFNA. For unadjusted geometric mean concentrations, see supplemental materials.

The remaining six PFAS (Sb-PFOA, PFDoA, Me-PFOSAA, PFOSA, PFBS, and PFHpA) did not meet the threshold of more than 50% of results above LOD and were analyzed for detectability as a binary outcome using logistic regression.

### Associations among occupations and PFAS concentrations

**Fig. 1:**
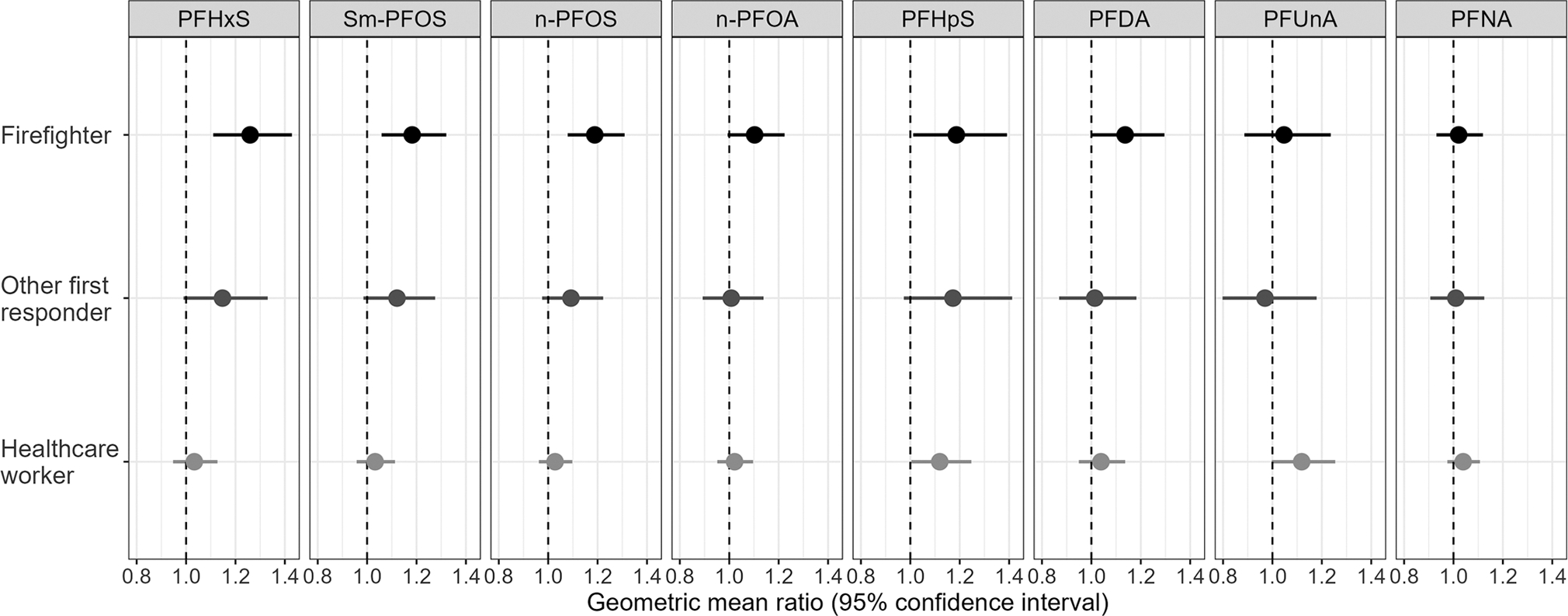
Geometric mean ratio estimates of per-and polyfluoroalkyl substance concentrations among firefighters, other first responders, and healthcare workers in Arizona during 2020–2023 relative to other essential workers adjusting for covariates and location. Linear regression results for log-transformed values. PFHxS perfluorohexanesulfonic acid; Sm-PFOS branched perfluorooctanesulfonic acid; n-PFOS linear perfluorooctanesulfonic acid; n-PFOA linear perfluorooctanoic acid; PFHpS perfluoroheptanesulfonic acid; PFDA perfluorodecanoic acid; PFUnA perfluoroundecanoic acid; PFNA perfluorononanoic acid.

Geometric mean ratios of PFAS serum concentrations by occupation were estimated using multilevel linear regression models for PFHxS, Sm-PFOS, n-PFOS, n-PFOA, PFHpS, PFDA, PFUnA, and PFNA. In all models, other essential workers were the referent occupational group. At enrollment, the adjusted geometric mean ratios (aGMR) and 95% CIs of four PFAS indicated higher concentrations among firefighters, compared with other essential workers, adjusting for covariates and location. These included PFHxS (aGMR: 1.26; 95% CI: 1.11–1.43), Sm-PFOS (aGMR: 1.18; 95% CI: 1.06–1.32), n-PFOS (aGMR: 1.19; 95% CI: 1.08–1.31), and PFHpS (aGMR: 1.19; 95% CI: 1.01–1.39) (Fig. 1; Supplementary Table 3). Both n-PFOA and PFDA were moderately elevated among firefighters, compared with other essential workers; however, 95% CIs included the null value of 1. Among other first responders, geometric mean concentrations of PFHxS, Sm-PFOS, and PFHpS were moderately elevated, compared with other essential workers; among healthcare workers, geometric mean concentrations of PFHpS and PFUnA were moderately elevated, compared with other essential workers. However, 95% CIs for these estimates among other first responders and healthcare workers included the null. Model results did not detect substantial differences in PFNA concentrations between any occupational group and other essential workers with estimates centered around the null. (Fig. 1) When stratified by sex, aGMR estimates for PFHxS, Sm-PFOS, n-PFOS, and PFHpS were higher among male participants among firefighters and other first responders compared with other essential workers. Among healthcare workers, aGMR estimates for Sm-PFOS, n-PFOA, PFHpS, PFUnA, and PFNA were higher among male participants, compared with other essential workers. Stratification by sex resulted in small sample sizes for some occupation-sex categories, results should be interpreted with caution (Supplementary Table 4).

**Fig. 2:**
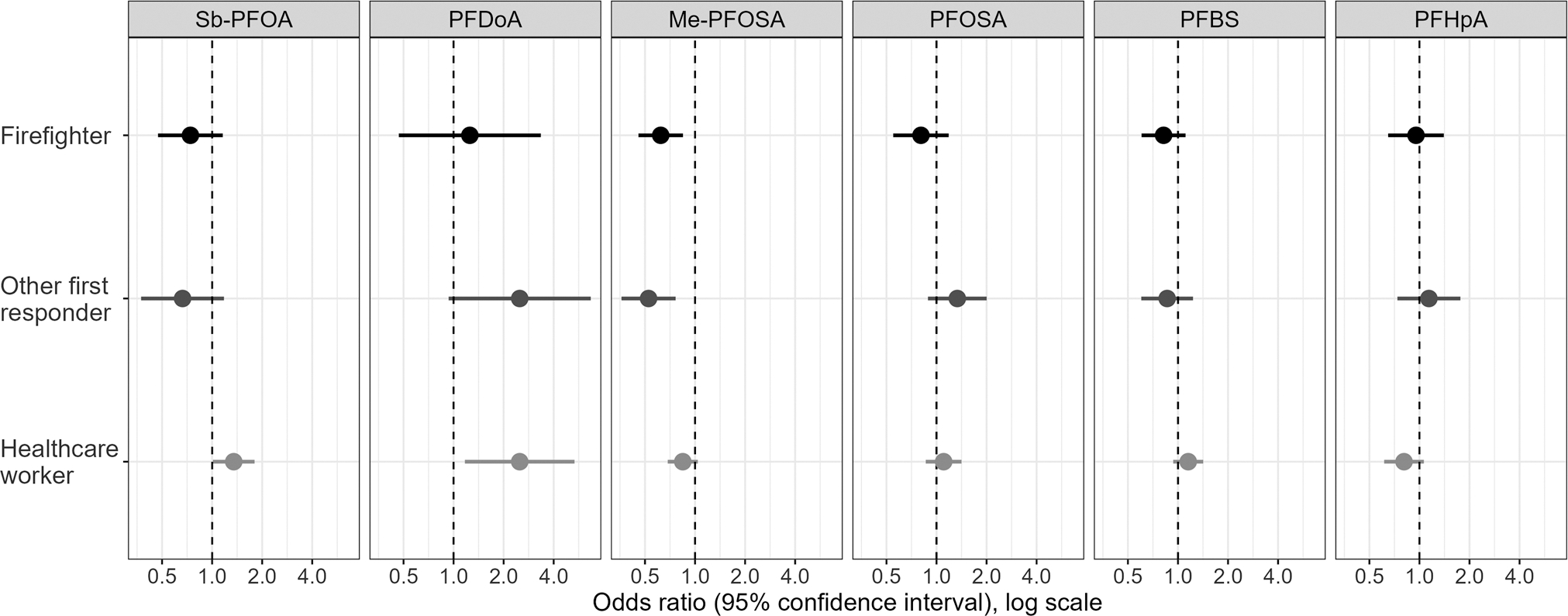
Odds of detection of per-and polyfluoroalkyl substance concentrations above the limit of detection among firefighters, other first responders, and healthcare workers in Arizona during 2020–2023 relative to other essential workers adjusting for covariates and location. Logistic regression results. Sb-PFOA branched perfluorooctanoic acid; PFDoA perfluorododecanoic acid; Me-PFOSA 2-(N-methylperfluorooctane sulfonamido) acetate; PFOSA perfluorooctanesulfonamide; PFBS perfluorobutanesulfonic acid; PFHpA perfluoroheptanoic acid.

Odds ratios for detection of PFAS values above the LOD by occupation were estimated using multilevel logistic regression models for Sb-PFOA, PFDoA, Me-PFOSAA, PFOSA, PFBA, and PFHpA and adjusted for the same variables as the linear regression models (Fig. 2; Supplementary Table 4). Among firefighters and other first responders, adjusted odds ratios (aOR) of detecting Me-PFOSAA were lower, compared with other essential workers (firefighter aOR: 0.62; 95% CI: 0.46–0.85; other first responder aOR: 0.53; 95% CI: 0.36–0.76) (Fig. 2). Among healthcare workers aOR of detecting Sb-PFOA and PFDoA were higher, compared with other essential workers (Sb-PFOA aOR: 1.35; 95% CI: 1.01–1.80; PFDoA aOR: 2.50; 95% CI: 1.17–5.34). When stratified by sex, aOR of detecting Sb-PFOA and Me-PFOSA was lower among male participants among firefighters and other first responders compared with other essential workers, and among healthcare workers, the aOR of detecting PFDoA was higher among female participants, compared with other essential workers. (Supplementary Table 6.)

#### Longitudinal associations among occupations and PFAS concentrations.

Multilevel linear regression models were used to estimate differences in eight PFAS serum concentrations by occupation over time, adjusting for covariates and participant-level correlation (Table 2). A total of 2857 observations across 1960 participants were available for PFHxS, PFHpS, PFDA, PFUnA, and PFNA. For Sm-PFOS, n-PFOS, and n-PFOA, 2605 observations across 1811 participants were available. Fixed effects for occupation aligned with estimates from cross-sectional models and indicated higher concentrations of PFHxS, Sm-PFOS, n-PFOS, n-PFOA, and PFHpS among firefighters, compared with other essential workers at the time of each participant’s first result. Concentrations of PFHpS were also higher among other first responders and healthcare workers, compared with other essential workers at the time of the first PFAS result. Interaction terms between each occupational group and time were close to the null value for seven of the eight PFAS analyzed and indicated no significant difference in the rate of change in PFAS concentrations between each occupation and other essential workers; only concentrations of PFUnA declined significantly more among other first responders, compared with other essential workers over time (aGMR: 0.85; 95% CI: 0.73–0.97). (Supplemental Fig. 1) Fixed effects for time indicated significant declines over time in concentrations of PFHxS (aGMR: 0.87; 95% CI: 0.85–0.90), Sm-PFOS (aGMR: 0.94; 95% CI: 0.92–0.96), n-PFOS (aGMR: 0.88; 95% CI: 0.86–0.90), n-PFOA (aGMR: 0.83; 95% CI: 0.81–0.85), and PFNA (aGMR: 0.88; 95% CI: 0.86–0.90) among other essential workers by year during the study period. Concentrations of PFDA and PFUnA also declined over time among other essential workers; however, the 95% CIs included the null value of one. Only PFHpS had no change in concentration detected at the timescale of this study. Longitudinal models showed a small indication of model inadequacy by a positive trend in the residuals. We explored alternate modeling strategies (splines for time, scaling of continuous variables, box-cox transformation of PFAS concentrations, and exclusion of observations with excess influence). None of these models resulted in a noticeably better model fit, nor did they change the inferential results for occupation and time.

## DISCUSSION

This analysis of PFAS serum concentrations by occupation in a large, prospective cohort of 1960 frontline workers in Arizona showed that firefighters had higher concentrations of PFHxS, Sm-PFOS, n-PFOS, and PFHpS, compared with other essential workers. Our study is the first to assess serum PFAS concentrations among healthcare workers and the first to identify moderate elevations of certain PFAS (PFHpS, PFUnA) and significantly higher odds of detection of Sb-PFOA and PFDoA among this group. We detected significant declines over time in PFAS concentrations during the 3-year study period of 6–17% per year among other essential workers for PFHxS, Sm-PFOS, n-PFOS, n-PFOA, and PFNA. A slightly greater decline in PFUnA over time was detected among other first responders, compared with other essential workers and no other interactions between PFAS concentrations and time were detected among other occupations when compared with other essential workers.

Among firefighters, our findings align with previous studies that demonstrated higher concentrations of PFHxS [15, 16, 21–24], PFHpS [16], and Sm-PFOS and n-PFOS [23], or total PFOS [16, 21, 22] in the United States and internationally. Contrary to other studies that detected higher concentrations of PFOA [21, 23–25] and PFNA [15, 23, 25] among firefighters, we did not detect significant elevations of n-PFOA or PFNA or differences in the odds of detecting Sb-PFOA among firefighters. Declines in the use and production of PFOA and PFNA and corresponding reductions in concentrations over time as these chemicals are removed from the body could explain this difference. We did not assess for sources of exposure to PFAS; however, pathways of exposure for firefighters in our study are likely to align with those for other municipal firefighters described by other studies [5, 13, 14]. Surprisingly, we found the detectability of Me-PFOSA to be lower among firefighters and other first responders, compared with other essential workers. The reasons for this difference are unknown. Among other first responders, moderate elevations in serum concentrations of PFHxS, Sm-PFOS, and PFHpS were similar to, although lower than, elevations detected among firefighters, compared with other essential workers. Our findings align with a focused evaluation of Maui County first responders that detected elevated median serum concentrations of PFHxS and PFOS among other first responders at similar concentrations to firefighters [34], although this study had a small sample size of other first responders and did not adjust for potential confounders.

We uniquely identified higher odds of detecting Sb-PFOA and PFDoA among healthcare workers, and moderately elevated concentrations of PFHpS and PFUnA, compared with other essential workers when adjusting for potential confounders and county of residence. PFAS exposure among healthcare workers has not been evaluated previously and potential sources for exposure are not well described. Certain personal protective equipment (PPE) and medical supplies contain PFAS, including single-use surgical masks [35], surgical gowns [11, 26], and radiograph film [26]. Increased occupation-related exposure to PFAS-containing PPE and other medical supplies might have a role in elevated PFAS among healthcare workers, although future studies are needed.

Our study had at least four limitations. First, we did not assess possible sources of PFAS exposure in this study and we were unable to control for nonoccupational sources of PFAS. Although drinking water is a notable source of PFAS exposure, the timing of our study did not align with either the 2013–2015 or 2023–2025 EPA unregulated contaminant monitoring rule (UCMR) drinking water quantification studies. Additionally, not all public water systems where participants in the present study resided were included in either UCMR study, further limiting possible adjustments for drinking water. A strength is our adjustment for county-level clustering in our analyses, which would have limited bias introduced by differences in county-level regulations of PFAS use and water treatment policies. Second, our study did not evaluate duration of employment and we were unable to account for differences in the length of occupation-associated exposure to PFAS. We also did not evaluate or control for firefighter types, alcohol consumption, or smoking status which could be potential confounders. Third, enrollment of participants in AZ HEROES and selection into the PFAS analytic cohort was not designed to be representative of the Arizona population and might have limited generalizability to other populations. Finally, we only evaluated 14 PFAS outcomes. Although thousands of other PFAS present possible exposures, we did not measure for other PFAS or have sufficient detectable values to evaluate other PFAS in this study.

PFAS exposure and bioaccumulation [3] have been associated with adverse health outcomes. These include increased risk for testicular and kidney cancers, increases in cholesterol levels, lower antibody response to some immunizations, pregnancy-induced hypertension, and preeclampsia, and small decreases in infant birth weight [7, 8]. Firefighters have been shown to have elevated risk for several cancers [36] and cardiovascular disease [37]. Potential additive effects of increased PFAS concentrations among firefighters and other occupation-associated health effects remain a concern. Procedures to limit contact with PFAS-containing foams and turnout gear [5, 13–15], enhanced and timely washing of gear contaminated with fire debris [5, 13, 17], and filtration of drinking water [6, 13] might be considered as interventions to reduce exposure to PFAS.

Among healthcare workers and other first responders, clinical effects of occupational exposure to PFAS have yet to be described. Interventions to generally reduce or eliminate contact with PFAS-containing materials and ingestion of PFAS-contaminated food and water might be considered. Although sources of specific PFAS exposure among healthcare workers are poorly characterized, they potentially include inhalation, ingestion, and dermal exposure; similar interventions for water filtration [38] and limiting contact with PFAS-containing water-resistant PPE could be used preemptively.

## CONCLUSION

Our detection of significantly elevated concentrations of certain PFAS among firefighters, compared with a more population-representative group of essential workers, implies distinct sources of occupational PFAS exposure remain for firefighters in Arizona. Additionally, we detected significantly higher odds of Sb-PFOA and PFDoA, and moderate elevations of other PFAS concentrations, among healthcare workers, compared with other representative workers. This finding raises concerns regarding potential unique sources of PFAS exposure among healthcare workers that have previously been unrecognized. Further characterization of sources of PFAS exposure for firefighters and healthcare workers is needed to guide interventions and further reduce exposure.

*45 C.F.R. part 46.102(l)(2), 21C.F.R. part 56; 42 U.S.C. Sect. 241(d); 5 U.S.C. Sect. 552a; 44 U.S.C. Sect. 3501 et seq.

## Supplementary Material

Supplement

## Figures and Tables

**Table 1.** Demographic and study characteristics of participants in the Arizona Healthcare, Emergency Responder, and Other Essential Worker Study with per-and polyfluoroalkyl substance measurements for at least one serum sample during 2020–2023.

	Overall	Firefighter	Other first responder	Healthcare Worker^a^	Other essential worker^b^
**n**	1960	280	159	787	734
**Year of initial blood draw** [*n* (%)]					
2020	221 (11.3)	39 (13.9)	27 (17.0)	101 (12.8)	54 (7.4)
2021	849 (43.3)	86 (30.7)	61 (38.4)	374 (47.5)	328 (44.7)
2022	290 (14.8)	43 (15.4)	27 (17.0)	104 (13.2)	116 (15.8)
2023	600 (30.6)	112 (40.0)	44 (27.7)	208 (26.4)	236 (32.2)
**>1 blood draw** (%)^c^	693 (35.4)	62 (22.1)	57 (35.8)	297 (37.7)	277 (37.7)
**Female** (%)	1233 (62.9)	50 (17.9)	69 (43.4)	619 (78.7)	495 (67.4)
**Age** (median [IQR])	46 [38,55]	44 [37,50]	44 [39,52]	45 [38,55]	50 [40,59]
**Race and ethnicity** (%)					
Hispanic	423 (21.6)	60 (21.4)	49 (30.8)	158 (20.1)	156 (21.3)
NH, White	1418 (72.3)	209 (74.6)	101 (63.5)	572 (72.7)	536 (73.0)
NH, Other^d^	119 (6.0)	11 (3.9)	9 (5.6)	57 (7.2)	42 (5.7)
**County of residence** (%)					
Pima	1184 (60.4)	154 (55.0)	96 (60.4)	438 (55.7)	496 (67.6)
Maricopa	492 (25.1)	79 (28.2)	25 (15.7)	222 (28.2)	166 (22.6)
Other	284 (14.5)	47 (16.8)	38 (23.9)	127 (16.1)	72 (9.8)
**Urban zip code** (%)	1878 (95.8)	263 (93.9)	145 (91.2)	758 (96.3)	712 (97.0)

*IQR* interquartile range; *NH* non-Hispanic. Percentages are in reference to column total in the first row (*n*).

a Healthcare worker occupations included inpatient, outpatient, or residential settings.

b Includes frontline and other essential workers in retail, education, utilities, and/or government occupations.

c Number of specimens collected and measured for PFAS per participant ranged from 1 to 8.

d Other race includes: Black or African American, American Indian, Native Hawaiian, Pacific Islander, Asian; NH, non-Hispanic ethnicity

**Table 2.** Longitudinal geometric mean ratio estimates of per-and polyfluoroalkyl substance concentrations over time among firefighters, other first responders, and healthcare workers relative to other essential workers in Arizona during 2020–2023.

	PFHxS (*n* = 2857)	Sm-PFOS (*n* = 2605)	n-PFOS (*n* = 2605)	n-PFOA (*n* = 2605)	PFHpS (*n* = 2857)	PFDA (*n* = 2857)	PFUnA (*n* = 2857)	PFNA (*n* = 2857)
*Predictors*	*Estimate (95% CI)*	*Estimate (95% CI)*	*Estimate (95% CI)*	*Estimate (95% CI)*	*Estimate (95% CI)*	*Estimate (95% CI)*	*Estimate (95% CI)*	*Estimate (95% CI)*
Firefighters	**1.26 (1.11–1.43)**	**1.18 (1.06–1.32)**	**1.19 (1.08–1.31)**	**1.12 (1.01–1.25)**	**1.20 (1.03–1.41)**	1.14 (1.00–1.30)	1.06 (0.90–1.25)	1.02 (0.93–1.12)
Other first responders	1.15 (1.00–1.34)	1.12 (0.98–1.27)	1.09 (0.97–1.22)	1.03 (0.91–1.16)	**1.21 (1.01–1.45)**	1.03 (0.88–1.19)	0.97 (0.80–1.18)	1.01 (0.91–1.12)
Flealthcare workers	1.05 (0.96–1.14)	1.04 (0.96–1.12)	1.03 (0.97–1.10)	1.03 (0.96–1.11)	**1.12 (1.01–1.25)**	1.05 (0.96–1.15)	1.12 (1.00–1.25)	1.04 (0.97–1.10)
Time (in years)	**0.87 (0.85–0.90)**	**0.94 (0.92–0.96)**	**0.88 (0.86–0.90)**	**0.83 (0.81–0.85)**	1.03 (0.96–1.11)	0.96 (0.91–1.01)	0.94 (0.88–1.00)	**0.88 (0.86–0.90)**
Time x Firefighters	0.99 (0.94–1.05)	0.97 (0.91–1.03)	1.01 (0.95–1.07)	0.97 (0.91–1.04)	0.99 (0.83–1.18)	1.05 (0.93–1.19)	1.01 (0.88–1.16)	1.01 (0.96–1.07)
Time x Other first responders	1.00 (0.94–1.06)	1.01 (0.95–1.07)	0.99 (0.94–1.05)	0.99 (0.92–1.06)	1.05 (0.87–1.25)	0.88 (0.78–1.00)	**0.85 (0.73–0.97)**	1.01 (0.96–1.07)
Time x Flealthcare workers	1.00 (0.96–1.03)	0.99 (0.96–1.03)	1.00 (0.97–1.03)	1.01 (0.98–1.05)	0.96 (0.87–1.06)	0.99 (0.92–1.07)	1.02 (0.94–1.10)	1.01 (0.98–1.04)

Note: Models included an interaction term between time (in years) and each level of occupation, adjusting for covariates and allowing for correlation between data points from the same person over time. Model was fit to log-transformed values; presented estimates are back-transformed. Covariate adjustment set included fixed effects of age, sex, race and ethnicity, year of measurement, county of residence, and a random intercept per person. All comparisons were among listed groups and other essential and frontline workers. Bold indicates statistically significant result based on *P* value < 0.05. *P* values are based on the Kenward-Roger approximation. PFHxS, perfluorohexanesulfonic acid; Sm-PFOS, branched perfluorooctanesulfonic acid; n-PFOS, linear perfluorooctanesulfonic acid; n-PFOA, linear perfluorooctanoic acid; PFHpS, perfluoroheptanesulfonic acid; PFDA, perfluorodecanoic acid; PFUnA, perfluoroundecanoic acid; PFNA, perfluorononanoic acid.

## Data Availability

Data are available from the corresponding author upon request.
