# Supplementary material for: Differences in serum concentrations of per-and polyfluoroalkyl substances by occupation among firefighters, other first responders, healthcare workers, and other essential workers in Arizona, 2020–2023
Source: J Expo Sci Environ Epidemiol. 2025 Mar 6;35(3):437–44. doi: 10.1038/s41370-025-00753-7 (PMC12068973; doi:10.1038/s41370-025-00753-7)
Supplement: Supplementary file 1 — Supplementary material [file 41370_2025_753_MOESM1_ESM.docx]

**Supplemental Material**

Differences in Serum Concentrations of Per-and Polyfluoroalkyl Substances by Occupation Among Firefighters, Other First Responders, Healthcare Workers, and Other Essential Workers in Arizona, 2020–2023

**Supplemental Table 1.** Proportion of samples above the limit of detection (n>LOD/total n) for each per-and polyfluoroalkyl substance among participants in the Arizona Healthcare, Emergency Responder, and Other Essential Worker Study during 2020–2023.

| **PFAS** | **Total** | **Firefighter** | **Other first responder** | **Healthcare worker** | **Other essential worker** | **Limit of Detection^a^** |
| --- | --- | --- | --- | --- | --- | --- |
| **Continuous** |  |  |  |  |  |  |
| PFHxS | 0.997 (1954/1960) | 1 (280/280) | 0.994 (158/159) | 0.997 (785/787) | 0.996 (731/734) | 0.019, 0.012, 0.012 |
| Sm-PFOS | 0.997 (1806/1811) | 0.996 (253/254) | 0.993 (144/145) | 1 (715/715) | 0.996 (694/697) | NA, 0.009, 0.009 |
| n-PFOS | 0.997 (1806/1811) | 0.996 (253/254) | 0.993 (144/145) | 0.999 (714/715) | 0.997 (695/697) | NA, 0.050, 0.047 |
| n-PFOA | 0.996 (1803/1811) | 0.996 (253/254) | 0.979 (142/145) | 0.999 (714/715) | 0.996 (694/697) | NA, 0.019, 0.018 |
| PFHpS | 0.913 (1654/1811) | 0.972 (247/254) | 0.910 (132/145) | 0.901 (644/715) | 0.905 (631/697) | NA, 0.005, 0.005 |
| PFDA | 0.692 (1357/1960) | 0.743 (208/280) | 0.692 (110/159) | 0.695 (547/787) | 0.67 (492/734) | 0.019, 0.031, 0.030 |
| PFUnA | 0.669 (1311/1960) | 0.646 (181/280) | 0.654 (104/159) | 0.696 (548/787) | 0.651 (478/734) | 0.006, 0.012, 0.012 |
| PFNA | 0.994 (1949/1960) | 0.993 (278/280) | 0.981 (156/159) | 0.997 (785/787) | 0.995 (730/734) | 0.008, 0.020, 0.020 |
| **Binary** |  |  |  |  |  |  |
| Sb-PFOA | 0.161 (292/1811) | 0.142 (36/254) | 0.110 (16/145) | 0.186 (133/715) | 0.154 (107/697) | NA, 0.027, 0.025 |
| PFDoA | 0.323 (80/248) | 0.256 (10/39) | 0.400 (12/30) | 0.398 (45/113) | 0.197 (13/66) | 0.013, NA, NA |
| Me-PFOSA | 0.407 (797/1960) | 0.354 (99/280) | 0.308 (49/159) | 0.402 (316/787) | 0.454 (333/734) | 0.005, 0.006, 0.006 |
| PFOSA | 0.222 (435/1960) | 0.175 (49/280) | 0.258 (41/159) | 0.233 (183/787) | 0.221 (162/734) | 0.004, 0.007, 0.006 |
| PFBS | 0.473 (928/1960) | 0.404 (113/280) | 0.428 (68/159) | 0.510 (401/787) | 0.471 (346/734) | 0.009, 0.010, 0.009 |
| PFHpA | 0.178 (349/1960) | 0.186 (52/280) | 0.214 (34/159) | 0.161 (127/787) | 0.185 (136/734) | 0.011, 0.034, 0.032 |

Note: PFHxS, perfluorohexanesulfonic acid; Sm-PFOS, branched perfluorooctanesulfonic acid; n-PFOS, linear perfluorooctanesulfonic acid; n-PFOA, linear perfluorooctanoic acid; PFHpS, perfluoroheptanesulfonic acid; PFDA, perfluorodecanoic acid; PFUnA, perfluoroundecanoic acid; PFNA, perfluorononanoic acid; Sb-PFOA, branched perfluorooctanoic acid; PFDoA, perfluorododecanoic acid; Me-PFOSA, 2-(N-methyl-perfluorooctane sulfonamido) acetate; PFOSA, perfluorooctanesulfonamide; PFBS, perfluorobutanesulfonic acid; PFHpA, perfluoroheptanoic acid; NA, not applicable.

**^a^**Limit of detection (LOD) values for certain PFAS assays changed over time. LOD values are ordered by time period as follows: May 2021–September 2022; October 2022–November 2022; December 2022–September 2023. NA indicates analyte was not measured during the corresponding time period.

***Absolute Geometric Mean PFAS Concentrations***

Crude geometric mean serum PFAS concentrations and 95% CIs for all PFAS with more than 50% of results above the LOD were estimated by occupational group of firefighters, other first responders, healthcare workers, and other essential workers. PFAS serum concentrations from adults in the most recent National Health and Nutrition Examination Survey (NHANES) for 2017–2018(1) were included as a referent comparison group representative of the U.S. population. Unadjusted geometric mean concentrations of all eight PFAS that were analyzed in our study as continuous measures were low, compared with NHANES. Only among firefighters were unadjusted concentrations of PFHxS and Sm-PFOS higher in our study, compared with NHANES 2017–2018 estimates. We expect lower concentrations to be related to the recent timing of our study (2020–2023) aligning with reductions in production and use of many PFAS. Industry shifts from long-chain PFAS (e.g., PFOA, PFOS, and PFHxS) to short-chain PFAS (e.g., PFBA) have also shifted PFAS elimination half-lives from a scale of years for long-chain PFAS to days for short chain PFAS,(2, 3) reducing the probability of detection after discrete exposure events. Despite low concentrations of PFAS in our overall study population, we detected significantly elevated concentrations of certain PFAS among firefighters, compared with a more population-representative group of workers.

**Supplemental table 2.** Unadjusted geometric mean and 95% CI estimates for PFAS serum concentrations among participants in the Arizona Healthcare, Emergency Responder, and Other Essential Worker Study during 2020–2023 with per-and polyfluoroalkyl substance measurements at enrollment by occupational group, in comparison with National Health and Nutrition Examination Survey (NHANES) 2017–2018 serum concentrations.

|  |  | Overall | Firefighter | Other first responder | Healthcare worker | Other essential worker | NHANES 2017─2018 |
| --- | --- | --- | --- | --- | --- | --- | --- |
|  | n | GM (95% CI) | GM (95% CI) | GM (95% CI) | GM (95% CI) | GM (95% CI) | GM (95% CI) |
| PFHxS | 1960 | 0.82 (0.78, 0.85) | 1.29 (1.16, 1.43) | 1.01 (0.86, 1.19) | 0.69 (0.65, 0.74) | 0.79 (0.73, 0.84) | 1.11 (1.05, 1.18) |
| Sm-PFOS | 1811 | 1.15 (1.11, 1.20) | 1.71 (1.55, 1.88) | 1.35 (1.16, 1.57) | 1.00 (0.95, 1.06) | 1.11 (1.05, 1.19) | 1.29 (1.21, 1.36) |
| n-PFOS | 1811 | 1.46 (1.41, 1.51) | 2.01 (1.85, 2.19) | 1.63 (1.44, 1.85) | 1.31 (1.25, 1.38) | 1.41 (1.33, 1.48) | 3.08 (2.92, 3.24) |
| n-PFOA | 1811 | 0.76 (0.73, 0.78) | 0.88 (0.82, 0.95) | 0.76 (0.66, 0.87) | 0.72 (0.69, 0.76) | 0.75 (0.71, 0.8) | 1.35 (1.29, 1.42) |
| PFHpS | 1811 | 0.06 (0.06, 0.06) | 0.11 (0.09, 0.12) | 0.08 (0.06, 0.09) | 0.05 (0.05, 0.06) | 0.06 (0.05, 0.06) | 0.23 (0.22, 0.24) |
| PFDA | 1960 | 0.06 (0.06, 0.06) | 0.06 (0.06, 0.07) | 0.06 (0.05, 0.06) | 0.06 (0.05, 0.06) | 0.06 (0.05, 0.06) | 0.20 (0.19, 0.21) |
| PFUnA | 1960 | 0.03 (0.03, 0.03) | 0.03 (0.02, 0.03) | 0.02 (0.02, 0.03) | 0.03 (0.03, 0.03) | 0.03 (0.02, 0.03) | 0.13 (0.12, 0.13) |
| PFNA | 1960 | 0.24 (0.23, 0.25) | 0.25 (0.24, 0.27) | 0.24 (0.22, 0.27) | 0.24 (0.23, 0.25) | 0.24 (0.23, 0.25) | 0.41 (0.39, 0.44) |

*Note.* GM, geometric mean serum concentration; PFHxS, perfluorohexanesulfonic acid; Sm-PFOS, branched perfluorooctanesulfonic acid; n-PFOS, linear perfluorooctanesulfonic acid; n-PFOA, linear perfluorooctanoic acid; PFHpS, perfluoroheptanesulfonic acid; PFDA, perfluorodecanoic acid; PFUnA, perfluoroundecanoic acid; PFNA, perfluorononanoic acid.

**Supplemental Table 3.** Geometric mean ratio estimates of per-and polyfluoroalkyl substance concentrations among firefighters, other first responders, and healthcare workers in Arizona during 2020–2023 relative to other essential workers adjusting for covariates and location. Linear regression results for log-transformed values.

|  | **PFHxS**  (*n* = 1,960) | **Sm-PFOS**  (*n* = 1,811) | **n-PFOS**  (*n* = 1,811) | **n-PFOA**  (*n* = 1,811) | **PFHpS**  (*n* = 1,811) | **PFDA**  (*n* = 1,960) | **PFUnA**  (*n* = 1,960) | **PFNA**  (*n* = 1,960) |
| --- | --- | --- | --- | --- | --- | --- | --- | --- |
| *Predictors* | *Estimate (95% CI)* | *Estimate*  *(95% CI)* | *Estimate (95% CI)* | *Estimate (95% CI)* | *Estimate (95% CI)* | *Estimate (95% CI)* | *Estimate (95% CI)* | *Estimate (95% CI)* |
| Firefighters | **1.26**  **(1.11–1.43)** | **1.18**  **(1.06–1.32)** | **1.19**  **(1.08–1.31)** | 1.10  (0.99–1.23) | **1.19**  **(1.01–1.39)** | 1.14  (1.00–1.30) | 1.05  (0.89–1.24) | 1.02  (0.93–1.12) |
| Other first responders | 1.15  (0.99–1.33) | 1.12  (0.98–1.28) | 1.09  (0.98–1.22) | 1.01  (0.89–1.14) | 1.17  (0.97–1.41) | 1.01  (0.87–1.18) | 0.97  (0.80–1.18) | 1.01  (0.91–1.13) |
| Healthcare workers | 1.03  (0.95–1.13) | 1.03  (0.96–1.11) | 1.03  (0.96–1.10) | 1.02  (0.95–1.10) | 1.12  (1.00–1.25) | 1.04  (0.95–1.14) | 1.12  (1.00–1.25) | 1.04  (0.97–1.11) |

*Note.* adjusted for fixed effects of age, sex, race and ethnicity, year of measurement, and including a random intercept per county of residence. All comparisons were among listed group and other essential and frontline workers. Bold indicates statistically significant result based on *P* value <0.05. *P* values estimated with a Kenward-Roger approximation. PFHxS, perfluorohexanesulfonic acid; Sm-PFOS, branched perfluorooctanesulfonic acid; n-PFOS, linear perfluorooctanesulfonic acid; n-PFOA, linear perfluorooctanoic acid; PFHpS, perfluoroheptanesulfonic acid; PFDA, perfluorodecanoic acid; PFUnA, perfluoroundecanoic acid; PFNA, perfluorononanoic acid.

**Supplemental Table 4.** Stratified geometric mean ratio estimates of per-and polyfluoroalkyl substance concentrations by sex among firefighters, other first responders, and healthcare workers in Arizona during 2020–2023 relative to other essential workers adjusting for covariates and location. Linear regression results for log-transformed values.

|  | **PFHxS** | **Sm-PFOS** | **n-PFOS** | **n-PFOA** | **PFHpS** | **PFDA** | **PFUnA** | **PFNA** |
| --- | --- | --- | --- | --- | --- | --- | --- | --- |
| *Predictors* | *Estimate (95% CI)* | *Estimate*  *(95% CI)* | *Estimate (95% CI)* | *Estimate (95% CI)* | *Estimate (95% CI)* | *Estimate (95% CI)* | *Estimate (95% CI)* | *Estimate (95% CI)* |
| Male participants | (*n* = 727) | (*n* = 665) | (*n* = 665) | (*n* = 665) | (*n* = 665) | (*n* = 727) | (*n* = 727) | (*n* = 727) |
| Firefighters (n = 230^a^) | **1.33 (1.14–1.56)** | **1.26 (1.10–1.45)** | **1.26 (1.12–1.42)** | 1.13 (1.00–1.27) | **1.23 (1.05–1.44)** | 1.16 (0.98–1.37) | 0.99 (0.80–1.21) | 1.07 (0.96–1.18) |
| Other first responders (n = 90^a^) | **1.27 (1.03–1.56)** | **1.25 (1.04–1.51)** | **1.21 (1.03–1.42)** | 1.06 (0.90–1.25) | **1.29 (1.04–1.60)** | 1.04 (0.84–1.29) | 0.96 (0.73–1.26) | 1.03 (0.89–1.18) |
| Healthcare workers (n = 168^a^) | 1.12 (0.94–1.32) | **1.18 (1.01–1.37)** | 1.14 (1.00–1.30) | **1.20 (1.05–1.37)** | **1.21 (1.02–1.44)** | 1.15 (0.95–1.38) | **1.30 (1.03–1.64)** | **1.19 (1.05–1.33)** |
| Female participants | (*n* = 1,233) | (*n* = 1,146) | (*n* = 1,146) | (*n* = 1,146) | (*n* = 1,146) | (*n* = 1,233) | (*n* = 1,233) | (*n* = 1,233) |
| Firefighters (n = 50^b^) | 1.07 (0.83–1.37) | 1.05 (0.84–1.31) | 1.02 (0.84–1.24) | 1.13 (0.90–1.41) | 1.13 (0.79–1.60) | 1.09 (0.84–1.41) | 1.31 (0.94–1.84) | 0.94 (0.77–1.13) |
| Other first responders (n = 69^b^) | 1.05 (0.85–1.31) | 1.03 (0.86–1.24) | 1.02 (0.87–1.20) | 0.97 (0.81–1.16) | 1.09 (0.81–1.45) | 0.99 (0.79–1.24) | 0.95 (0.72–1.27) | 1.02 (0.87–1.20) |
| Healthcare workers (n = 619^b^) | 0.99 (0.90–1.10) | 0.99 (0.90–1.07) | 0.99 (0.92–1.07) | 0.98 (0.90–1.06) | 1.09 (0.95–1.25) | 1.01 (0.91–1.12) | 1.08 (0.95–1.24) | 0.99 (0.92–1.07) |

^a^Counts for male participants for PFHxS, PFDA, PFUnA, and PFNA. Counts for male participants for Sm-PFOS, n-PFOS, and PFHpS were 210 firefighters, 79 other first responders, and 152 healthcare workers.

^b^Counts for female participants for PFHxS, PFDA, PFUnA, and PFNA. Counts for female participants for Sm-PFOS, n-PFOS, and PFHpS were 44 firefighters, 66 other first responders, and 563 healthcare workers.

*Note.* Stratified by sex, adjusted for fixed effects of age, race and ethnicity, year of measurement, and including a random intercept per county of residence. All comparisons were among listed group and other essential and frontline workers. Bold indicates statistically significant result based on *P* value <0.05. *P* values estimated with a Kenward-Roger approximation. PFHxS, perfluorohexanesulfonic acid; Sm-PFOS, branched perfluorooctanesulfonic acid; n-PFOS, linear perfluorooctanesulfonic acid; n-PFOA, linear perfluorooctanoic acid; PFHpS, perfluoroheptanesulfonic acid; PFDA, perfluorodecanoic acid; PFUnA, perfluoroundecanoic acid; PFNA, perfluorononanoic acid.

**Supplemental Table 5.** Odds of detection of per-and polyfluoroalkyl substance concentrations above the limit of detection among firefighters, other first responders, and healthcare workers in Arizona during 2020–2023 relative to other essential workers adjusting for covariates and location. Logistic regression results.

|  | **Sb-PFOA**  (*n* = 1,811) | **PFDoA**  (*n* = 248) | **Me-PFOSA**  (*n* = 1,960) | **PFOSA**  (*n* = 1,960) | **PFBS**  (*n* = 1,960) | **PFHpA**  (*n* = 1,960) |
| --- | --- | --- | --- | --- | --- | --- |
| *Predictors* | *Odds Ratio*  *(95% CI)* | *Odds Ratio*  *(95% CI)* | *Odds Ratio*  *(95% CI)* | *Odds Ratio*  *(95% CI)* | *Odds Ratio*  *(95% CI)* | *Odds Ratio*  *(95% CI)* |
| Firefighters | 0.74 (0.47–1.16) | 1.25 (0.47–3.35) | **0.62 (0.46–0.85)** | 0.81 (0.55–1.18) | 0.82 (0.60–1.11) | 0.95 (0.65–1.40) |
| Other first responders | 0.66 (0.37–1.18) | 2.50 (0.93–6.68) | **0.53 (0.36–0.76)** | 1.33 (0.89–2.00) | 0.86 (0.60–1.23) | 1.14 (0.74–1.76) |
| Healthcare workers | **1.35 (1.01–1.80)** | **2.50 (1.17–5.34)** | 0.84 (0.69–1.04) | 1.10 (0.86–1.41) | 1.15 (0.94–1.42) | 0.81 (0.61–1.06) |

*Note.* Bold indicates statistically significant result based on *P* value <0.05. Sb-PFOA, branched perfluorooctanoic acid; PFDoA, perfluorododecanoic acid; Me-PFOSA, 2-(N-methyl-perfluorooctane sulfonamido) acetate; PFOSA, perfluorooctanesulfonamide; PFBS, perfluorobutanesulfonic acid; PFHpA, perfluoroheptanoic acid.

**Supplemental Table 6.** Stratified odds of detection of per-and polyfluoroalkyl substance concentrations above the limit of detection by sex among firefighters, other first responders, and healthcare workers in Arizona during 2020–2023 relative to other essential workers adjusting for covariates and location. Logistic regression results.

|  | **Sb-PFOA** | **PFDoA** | **Me-PFOSA** | **PFOSA** | **PFBS** | **PFHpA** |
| --- | --- | --- | --- | --- | --- | --- |
| *Predictors* | *Odds Ratio*  *(95% CI)* | *Odds Ratio*  *(95% CI)* | *Odds Ratio*  *(95% CI)* | *Odds Ratio*  *(95% CI)* | *Odds Ratio*  *(95% CI)* | *Odds Ratio*  *(95% CI)* |
| Male participants | (*n* = 665) | (*n* = 102) | (*n* = 727) | (*n* = 727) | (*n* = 727) | (*n* = 727) |
| Firefighters (n = 230^a^) | **0.56 (0.32–0.96)** | 0.76 (0.21–2.73) | **0.57 (0.39–0.84)** | **0.60 (0.37–0.98)** | 0.71 (0.49–1.04) | 0.83 (0.52–1.33) |
| Other first responders (n = 90^a^) | **0.41 (0.17–0.97)** | 1.95 (0.55–6.92) | **0.43 (0.26–0.73)** | 1.10 (0.62–1.95) | 0.64 (0.38–1.06) | 0.80 (0.43–1.48) |
| Healthcare workers (n = 168^a^) | 1.37 (0.80–2.32) | 1.80 (0.52–6.24) | 0.78 (0.52–1.16) | 1.00 (0.62–1.62) | 1.05 (0.70–1.59) | 0.77 (0.47–1.27) |
| Female participants | (*n* = 1,146) | (*n* = 146) | (*n* = 1,233) | (*n* =1,233) | (*n* = 1,233) | (*n* = 1,233) |
| Firefighters (n = 50^b^) | 1.52 (0.66–3.50) | 2.56 (0.50–13.07) | 0.67 (0.35–1.25) | 1.72 (0.88–3.36) | 0.87 (0.48–1.59) | 1.36 (0.66–2.83) |
| Other first responders (n = 69^b^) | 1.02 (0.48–2.20) | 2.34 (0.43–12.58) | 0.67 (0.39–1.15) | 1.48 (0.82–2.67) | 1.10 (0.66–1.84) | 1.57 (0.85–2.91) |
| Healthcare workers (n = 619^b^) | 1.40 (0.99–1.98) | **3.32 (1.14–9.66)** | 0.88 (0.69–1.13) | 1.19 (0.89–1.59) | 1.19 (0.93–1.52) | 0.87 (0.62–1.21) |

^a^Counts for male participants for Me-PFOSA, PFOSA, PFBS, and PFHpA. Counts for male participants for Sb-PFOA, were 210 firefighters, 79 other first responders, and 152 healthcare workers, and for PFDoA were 28 firefighters, 20 other first responders, and 24 healthcare workers.

^b^Counts for female participants for Me-PFOSA, PFOSA, PFBS, and PFHpA. Counts for female participants for Sb-PFOA were 44 firefighters, 66 other first responders, and 563 healthcare workers, and for PFDoA were 11 firefighters, 10 other first responders, and 89 healthcare workers.

*Note.* Stratified by sex, adjusted for fixed effects of age, race and ethnicity, year of measurement, and including a random intercept per county of residence. Bold indicates statistically significant result based on *P* value <0.05. Sb-PFOA, branched perfluorooctanoic acid; PFDoA, perfluorododecanoic acid; Me-PFOSA, 2-(N-methyl-perfluorooctane sulfonamido) acetate; PFOSA, perfluorooctanesulfonamide; PFBS, perfluorobutanesulfonic acid; PFHpA, perfluoroheptanoic acid.

(A)
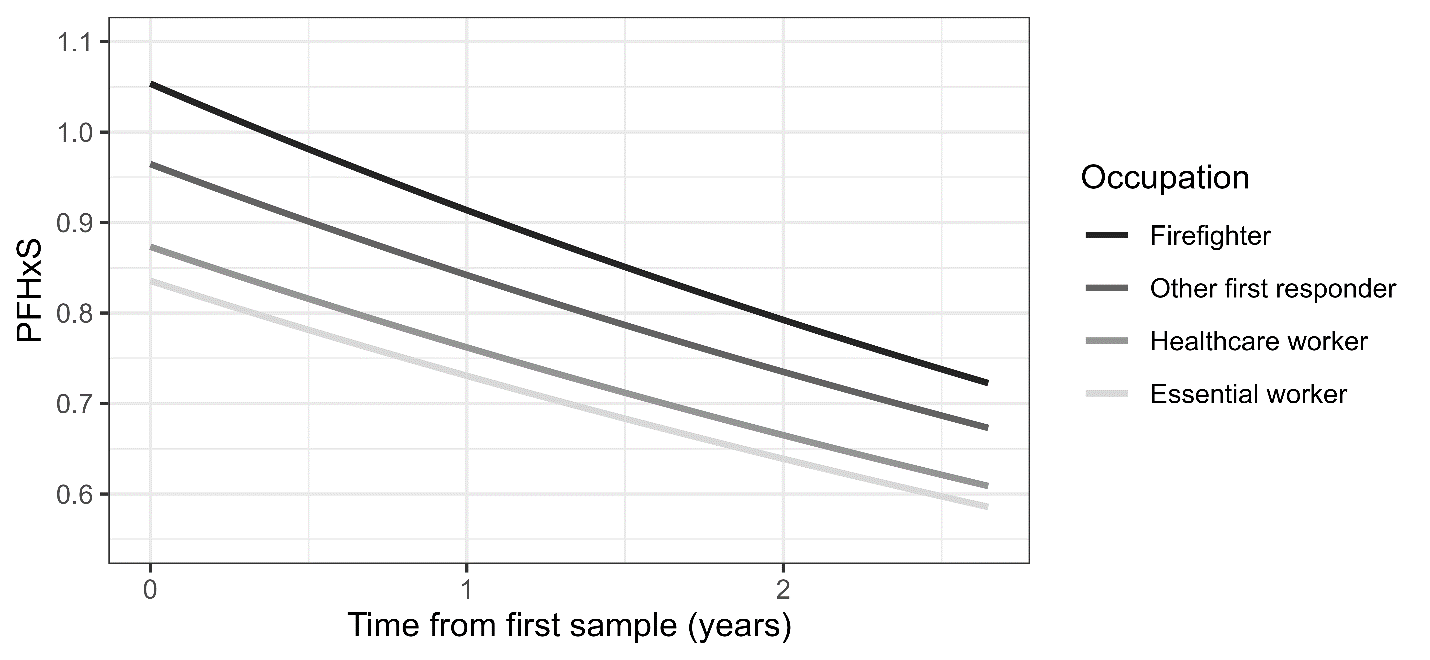


(B)
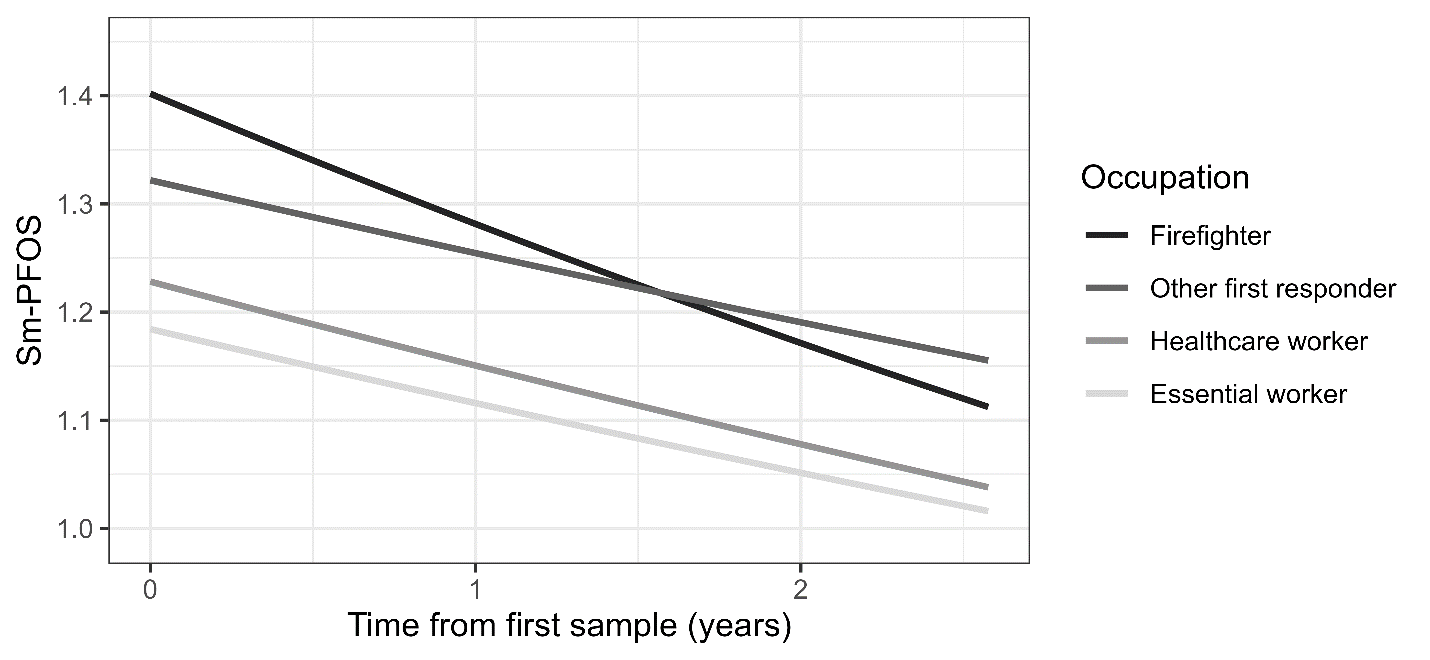


(C)
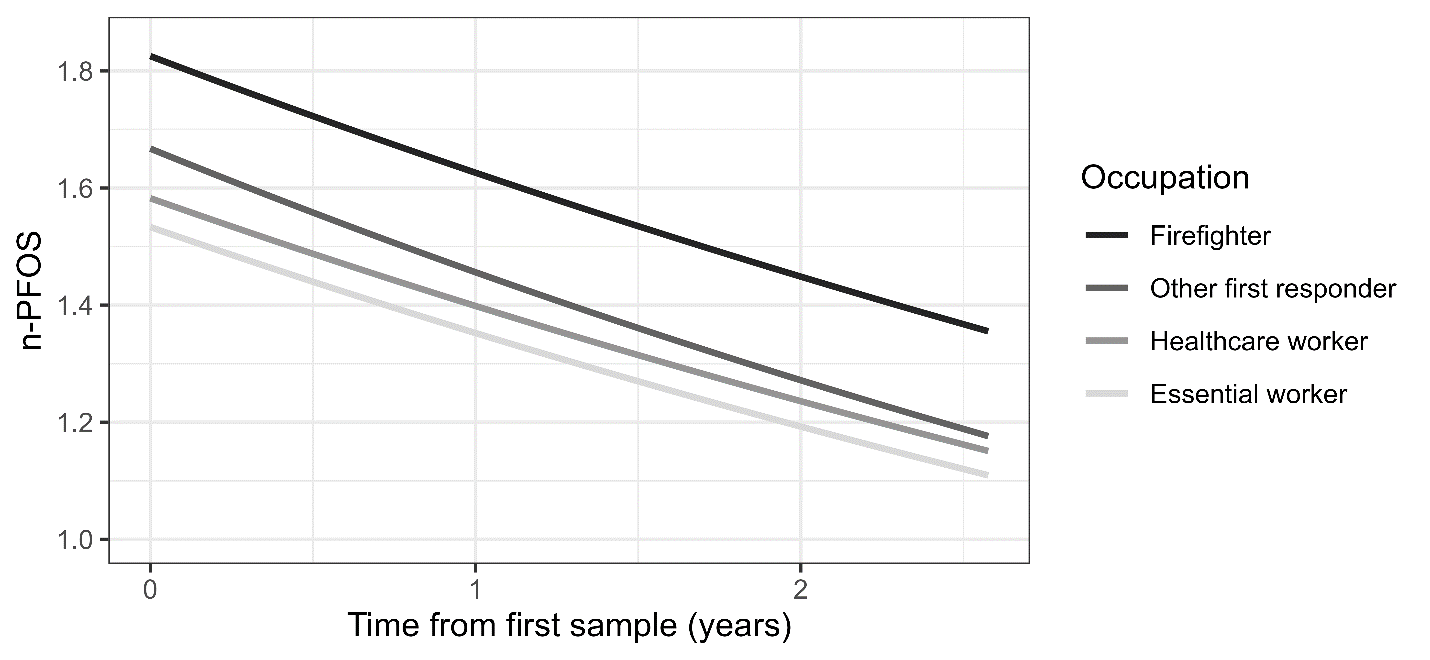


(D)
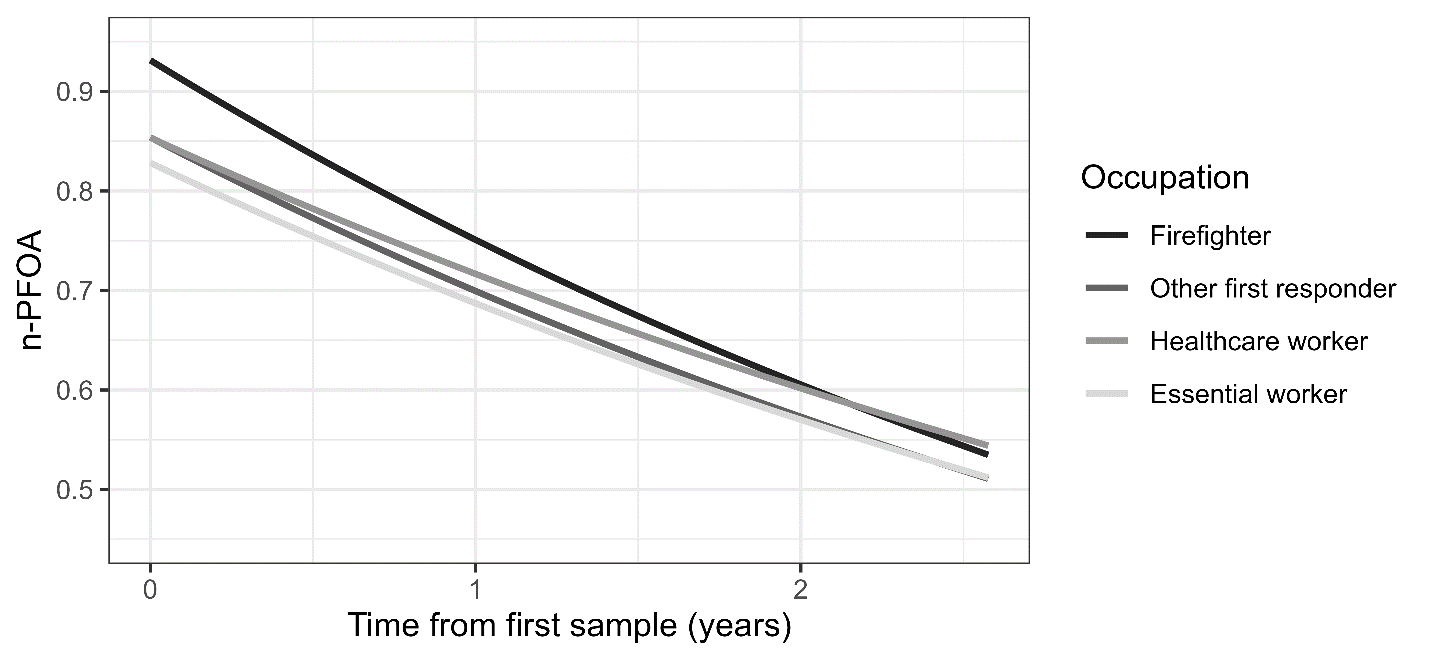


(E)
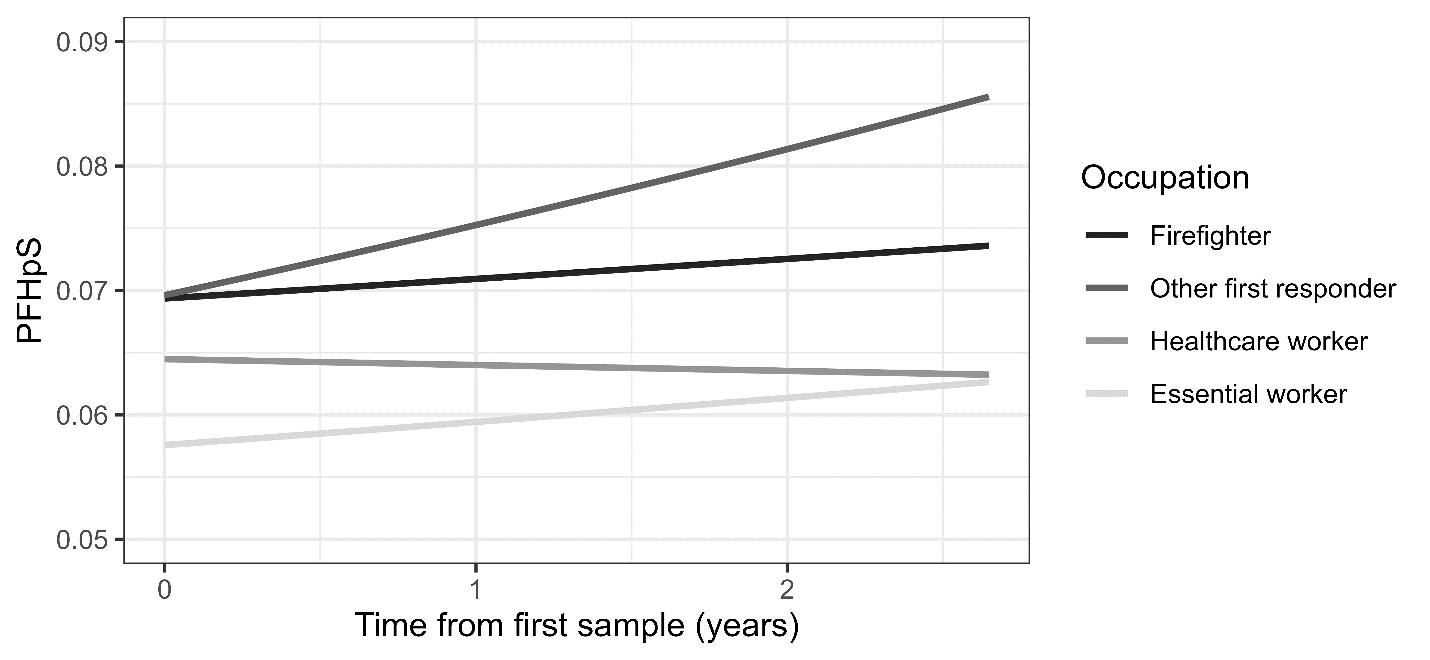


(F)
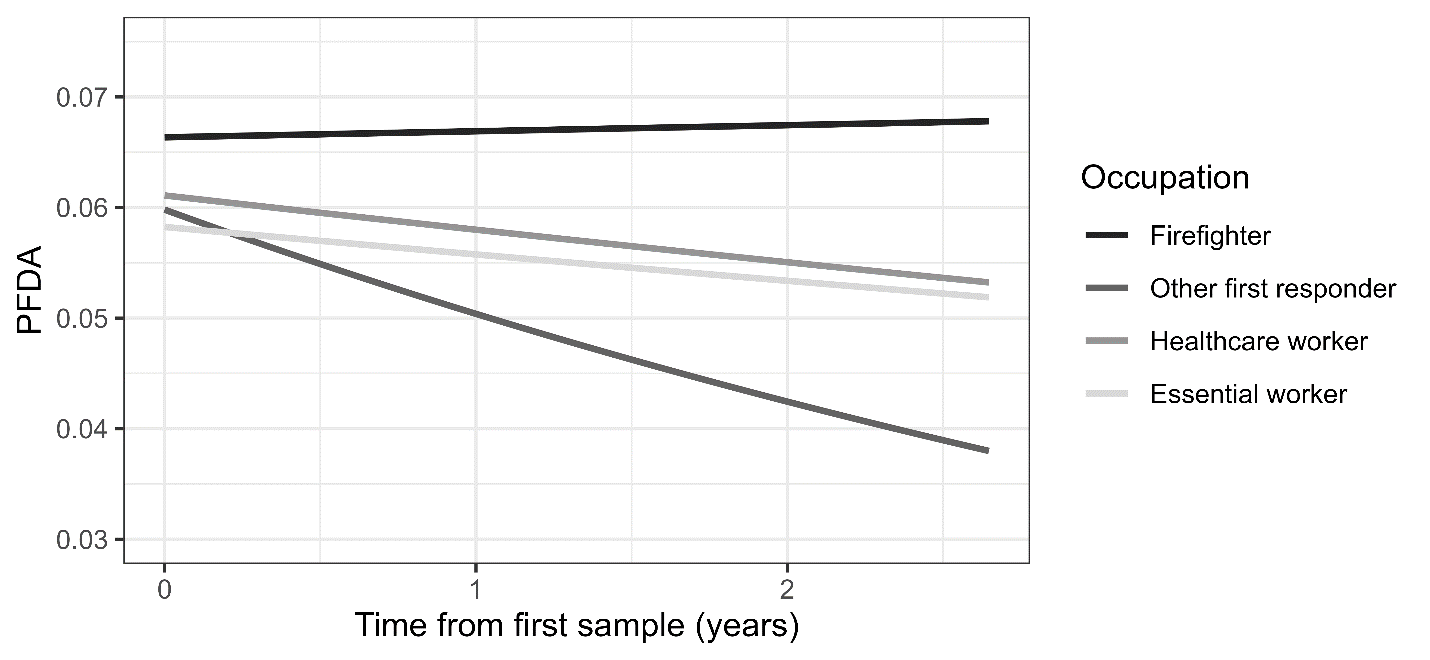


(G)
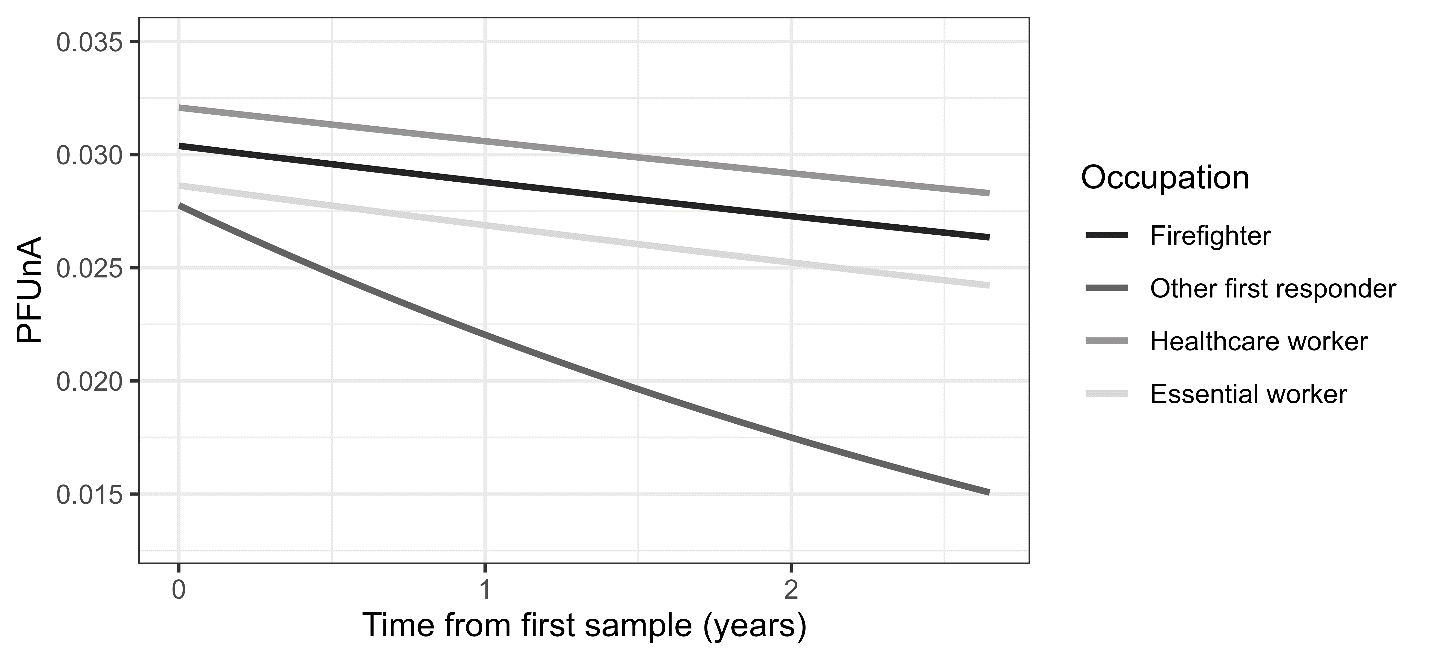


(H)
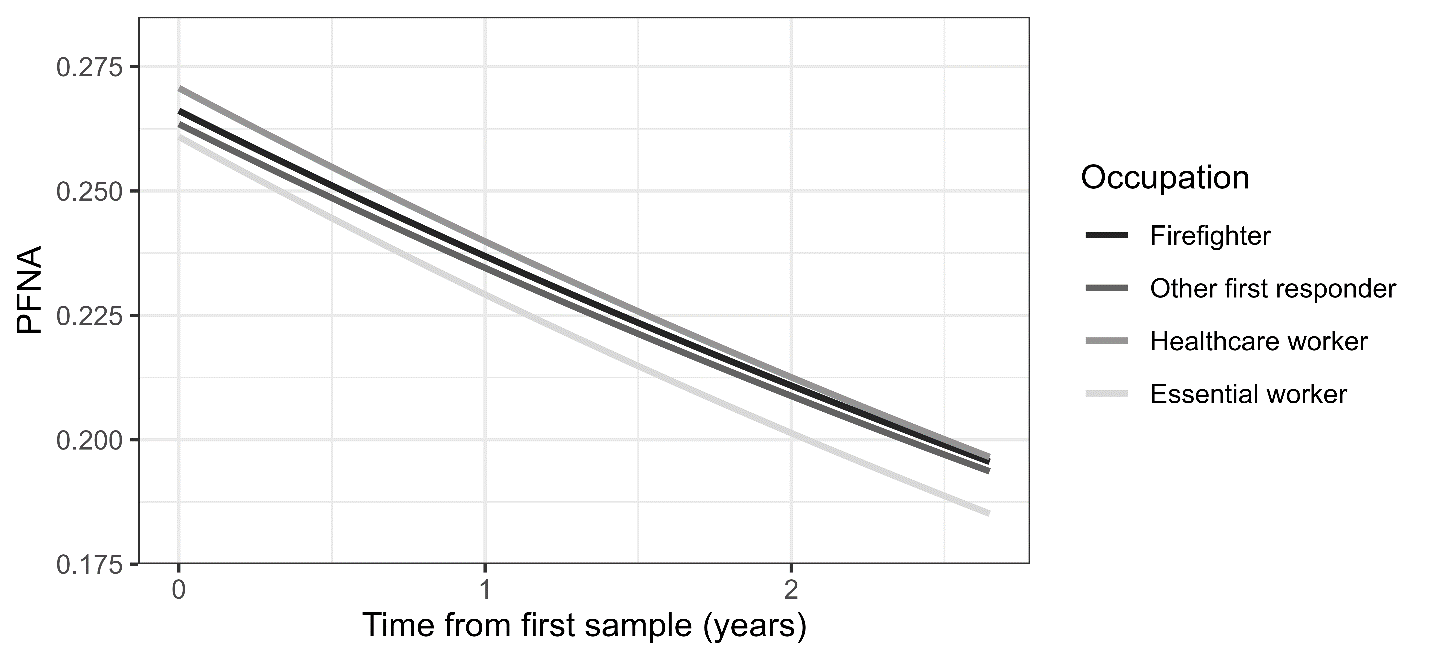


**Supplemental Figure 1.** Estimated longitudinal change in PFAS serum concentrations among firefighters, other first responders, healthcare workers, and other essential workers adjusting for covariates for (A) perfluorohexanesulfonic acid (PFHxS), (B) branched perfluorooctanesulfonic acid (Sm-PFOS), (C) linear perfluorooctanesulfonic acid (n-PFOS), (D) linear perfluorooctanoic acid (n-PFOA), (E) perfluoroheptanesulfonic acid (PFHpS), (F) perfluorodecanoic acid (PFDA), (G) perfluoroundecanoic acid (PFUnA), and (H) perfluorononanoic acid (PFNA). All models were fit to log-transformed values, plotted estimates are back-transformed. Note y-axis scale varies by PFAS to reflect PFAS-specific variation in the range of serum concentrations.

**References:**

1. Centers for Disease Control and Prevention (CDC). National Health and Nutrition Examination Survey Data. In: (NCHS) NCfHS, editor.: U.S. Department of Health and Human Services, Centers for Disease Control and Prevention.

2. Gaines LGT. Historical and current usage of per- and polyfluoroalkyl substances (PFAS): A literature review. Am J Ind Med. 2023;66(5):353-78.

3. Environmental Protection Agency (EPA). Multi-Industry Per-and Polyfluoroalkyl Substances (PFAS) Study–2021 Preliminary Report. Accessed on. 2021;25(04):2023.
